# Exosomal Liquid Biopsy for the Early Detection of Gastric Cancer: The DESTINEX Multicenter Study

**DOI:** 10.1001/jamasurg.2025.2493

**Published:** 2025-07-30

**Authors:** Silei Sui, Caiming Xu, Mitsuro Kanda, Yoshinaga Okugawa, Yuji Toiyama, Joon Oh Park, Hoon Hur, Song Cheol Kim, Akinobu Taketomi, Yasuhiro Kodera, Xiangdong Cheng, Man Li, Ajay Goel

**Affiliations:** 1 Department of Molecular Diagnostics and Experimental Therapeutics, Beckman Research Institute of City of Hope, Biomedical Research Center, Monrovia, California; 2 Department of Oncology, The Second Affiliated Hospital of Dalian Medical University, Dalian, China; 3 Department of General Surgery, The First Affiliated Hospital of Dalian Medical University, Dalian, China; 4 Department of Gastroenterological Surgery, Nagoya University Graduate School of Medicine, Nagoya, Japan; 5 Division of Reparative Medicine, Department of Gastrointestinal and Pediatric Surgery, Institute of Life Sciences, Mie University Graduate School of Medicine, Mie, Japan; 6 Division of Hematology-Oncology, Department of Medicine, Samsung Medical Center, Sungkyunkwan University School of Medicine, Seoul, South Korea; 7 Department of Surgery, Ajou University of School of Medicine, Suwon, Korea; 8 Cancer Biology Graduate Program, Ajou University Graduate School of Medicine, Suwon, Korea; 9 Division of Hepato-Biliary and Pancreatic Surgery, Department of Surgery, University of Ulsan College of Medicine, Asan Biomedical Engineering Research Center, AMIST, Asan Medical Center, Seoul, Korea; 10 Department of Gastroenterological Surgery I, Graduate School of Medicine, Hokkaido University, Hokkaido, Japan; 11 Department of Gastric Surgery, Zhejiang Cancer Hospital, Hangzhou, Zhejiang, China; 12 City of Hope Comprehensive Cancer Center, Duarte, California

## Abstract

**Importance:**

Gastric cancer (GC) is the third leading cause of cancer-related deaths worldwide, primarily attributed to delayed detection. The invasive and cost-prohibitive nature of endoscopy for GC screening highlights the urgent need for noninvasive biomarkers.

**Objective:**

To develop an exosome-based diagnostic signature to facilitate blood-based, early detection of patients with GC.

**Design, Setting, and Participants:**

This was a multicenter, population-based, retrospective, case-control study that analyzed specimens collected between January 1, 2016, and December 30, 2020. The study encompassed the discovery, training, validation, and evaluation phases of biomarker development. This study was conducted at 4 major referral centers: Nagoya University Hospital in Japan and Ajou University Hospital, Asan Medical Center, and Samsung Medical Center in South Korea, providing a broad representation of advanced clinical care settings. The study included patients with GC, classified according to the TNM (tumor-node-metastasis) classification (8th edition), and controls without disease. Key sociodemographic data, including age and sex, were recorded for all participants. Data were analyzed from October 2022 to July 2024.

**Exposures:**

Results were obtained from tissue and serum microRNA (miRNA) profiling and expression analysis. Frozen tissue collection and blood draws were conducted intraoperatively, preoperatively, and 3 months postoperatively.

**Main Outcomes and Measures:**

Diagnostic performance of GC detection using an exosome-based miRNA signature.

**Results:**

A total of 809 specimens from 480 patients (mean [SD] age, 61.9 [9.8] years; 336 male [70%]) in the training and validation cohorts were analyzed. A panel of 8 cell-free miRNAs and 10 exosomal miRNAs was initially developed in the discovery phase, which was subsequently reduced using machine learning algorithms to a panel of 8 cell-free and 9 exosomal miRNAs during the training phase. This 17-miRNA signature robustly identified GC with area under the curve (AUC) values of 96.3% (95% CI, 94.3%-98.4%) and 95.3% (95% CI, 92.8%-97.9%) in the training and validation cohorts, respectively. Additionally, 5 overlapping miRNAs were observed between cell-free and exosomal panels and exhibited a comparable efficacy in identifying patients with GC. Finally, we established a 10-miRNA signature (Destinex), which successfully identified early-stage (pT1) GC (AUC = 96.8%; 95% CI, 93.5%-100%). Finally, a significant decrease in miRNA expression levels in postsurgery serum specimens confirmed the robustness of the panel specificity.

**Conclusion and Relevance:**

Results of this case-control study suggest that the Destinex assay was robust for early detection of GC, highlighting its potential for clinical application in the noninvasive identification of GC.

## Key Points

**Question:**

What is the clinical utility of exosome-based microRNA as a liquid biopsy biomarker for early detection of gastric cancer?

**Findings:**

In this multicenter case-control study of 809 specimens from 480 patients in the training and validation cohorts, the 10-microRNA signature Destinex assay demonstrated robust performance for early detection of gastric cancer, achieving high area under the curve values in both the training and validation cohorts. For early-stage (pT1) gastric cancer, Destinex achieved a high sensitivity and specificity in distinguishing gastric cancer in those with the disease vs those without it.

**Meaning:**

These findings suggest that the Destinex assay could enhance the existing toolkit for gastric cancer screening, and its implementation is anticipated to improve patient outcomes significantly.

## Teaser

This case-control study investigates the clinical utility of exosome-based microRNA as a liquid biopsy biomarker for the early detection of gastric cancer.

## Introduction

Gastric cancer (GC) is the fifth most common cancer and the third leading cause of cancer-related deaths globally.^1,2^ Approximately 60% of patients with GC are diagnosed at an advanced stage, making them ineligible for curative treatment and leading to early metastasis and poor prognosis.^3^ Mass screening using photofluorography or endoscopy in individuals 40 years and older has been implemented in high-prevalence regions, such as Japan and Korea.^4,5^ Although these early detection efforts have improved the outcomes of patients with GC, they are impractical in low-prevalence countries due to their invasiveness and high costs. Noninvasive serological markers, including carcinoembryonic antigen, cancer antigen 19-9 (CA19-9), CA724, CA125, and α-fetoprotein, are inadequate for GC detection owing to low specificity and sensitivity, especially for early-stage cancers.^6^ Thus, there is an unmet clinical need for cost-effective, minimally invasive approaches for early GC detection.

Liquid biopsies, including circulating tumor cells (CTCs), circulating tumor DNA (ctDNA), RNAs, and exosomes, hold tremendous potential for cancer diagnosis, recurrence prediction, and therapeutic monitoring.^7,8,9,10^ MicroRNAs (miRNAs) are a class of noncoding RNAs that play a pivotal role in tumor progression.^11^ Their stability in blood and aberrant expression makes them promising biomarkers for cancer detection.^12,13,14,15^ Although circulating cell-free miRNAs are highly abundant and sensitive in bodily fluids, their specificity remains debatable, as they may derive not only from tumor cells but also from inflammatory and immune cells in the tumor microenvironment.^16^ Hence, there has been a desire to build on the promise of miRNAs and develop strategies for more cancer-specific analyses of these analytes as disease indicators.

Recent years have witnessed a burgeoning interest in studying exosomes, small extracellular vesicles crucial for intercellular communication.^17^ More importantly, exosomal cargo components (eg, DNA, RNA, proteins, metabolites, etc) have emerged as powerful and attractive analytes for developing cancer biomarkers.^18,19,20^ Tumor cells secrete approximately 10-fold more exosomes than normal cells, which exhibit unique and specific miRNA expression profiles,^21,22^ making exosomal miRNAs promising noninvasive biomarkers for cancer diagnosis.^18,23^ Considering that cell-free miRNAs offer high sensitivity whereas exosomal miRNAs offer tissue specificity, there has been an emerging viewpoint that combining the 2 might be optimal for developing effective biomarkers. In support of this hypothesis, our group recently provided compelling evidence that combined cell-free miRNA and exosomal miRNA signatures serve as robust, noninvasive biomarkers for early pancreatic cancer detection.^23^

In this study, we undertook a systematic genome-wide transcriptomic profiling effort to discover GC-specific biomarkers, followed by extensive bioinformatic analysis using machine-learning approaches to establish a novel cell-free and exosomal miRNA signature for early GC detection. After the initial biomarker discovery, this noninvasive diagnostic signature was trained and validated in independent, multinational cohorts of patients with GC. This enabled the development of Destinex, a noninvasive, inexpensive, and robust assay for early gastric neoplasia detection.

## Methods

### Study Design

This multicenter case-control study was approved by the ethics committee of all participating institutions—including the Asan Medical Center, Ajou University, Samsung Medical Center, Nagoya University, Hokkaido University, Mie University, and City of Hope. Written informed consent was obtained from all participants. This study followed the Strengthening the Reporting of Observational Studies in Epidemiology (STROBE) reporting guidelines.

Our study comprised 4 phases: (1) an initial comprehensive, genome-wide transcriptomic sequencing-based biomarker discovery phase using small RNA sequencing, (2) a serum-based biomarker training phase, (3) a subsequent validation phase, and (4) a diagnostic performance evaluation phase in an independent clinical cohort of patients with other gastrointestinal cancers, along with paired preoperative and postoperative serum samples from patients diagnosed with GC. All study participants self-reported Asian race. Details on patient cohorts and additional methods are provided in the eMethods in Supplement 1.

### Statistical Analysis

Categorical variables are described using number and percentage, and continuous variables are represented by R software, version 4.2.2 (R Foundation for Statistical Computing), and GraphPad Prism, version 10.4.1 (GraphPad Software). The differential expression analysis was performed using the limma package in R. To evaluate the performance of diagnostic biomarkers, the receiver operating characteristic curve (ROC) analysis was conducted using the pROC package. Area under the receiver operating characteristic curve (AUROC) with 95% CIs was computed by the method of DeLong, with optimal cutoff thresholds determined by the Youden index. The decision curve analysis (DCA) was developed to delineate the net benefit value of the miRNA signature by using the rmda function. Calibration curve analysis was applied to assess the calibration of the miRNA signature using the CalibrationCurves function in R. The Mann-Whitney *U* and *t*-test were applied to compare 2 independent groups with continuous variables. Additionally, the χ^2^ test was used to compare area under the curve (AUC) and sensitivity across different subgroups. A *P* value <.05 was considered a statistically significant change. Study data were analyzed from October 2022 to July 2024.

## Results

### Genome-Wide Transcriptional Profiling 

The initial comprehensive discovery phase of our study used small RNA sequencing of 189 specimens. The biomarker training phase included 263 specimens. The subsequent validation phase included analysis of 217 serum specimens, and the diagnostic performance evaluation phase included an independent clinical cohort of 100 patients with other GCs, along with 20 paired preoperative and postoperative serum samples from patients diagnosed with GC. A total of 809 specimens from 480 patients (mean [SD] age, 61.9 [9.8] years; 132 female [29%]; 336 male [70%]; 7 not available [1%]) in the training (263 patients) and validation (217 patients) cohorts were analyzed. The design and workflow are detailed in eFigure 1 in Supplement 1. The clinicopathological characteristics of each clinical cohort are described in eTable 1 in Supplement 1.

Our research aimed to identify cell-free– and exosomal-miRNA biomarkers for early GC detection. Toward this aim, we conducted systematic sequencing of large clinical cohorts, followed by rigorous bioinformatic analyses, to identify candidate miRNAs. First, differentially expressed analysis of small RNA-sequencing data from 47 primary GC and matched adjacent normal tissues identified 44 upregulated and 64 downregulated miRNAs (log_2_fold change [FC] >0.5; *P* < .01; the criteria listed represent our selection thresholds) (eFigure 2A in Supplement 1). Subsequently, similar analysis of exosomal and cell-free fractions from 32 exosomal RNAs and 43 cell-free RNAs yielded a panel of 54 upregulated and 72 downregulated exosomal miRNAs (log_2_FC >1; *P* < .01) (eFigure 2B in Supplement 1), and a panel of 112 upregulated and 52 downregulated cell-free miRNAs (log_2_FC >1; *P* < .01) (eFigure 2C in Supplement 1) that discriminated patients with GC from controls without disease.

**Figure 1.**
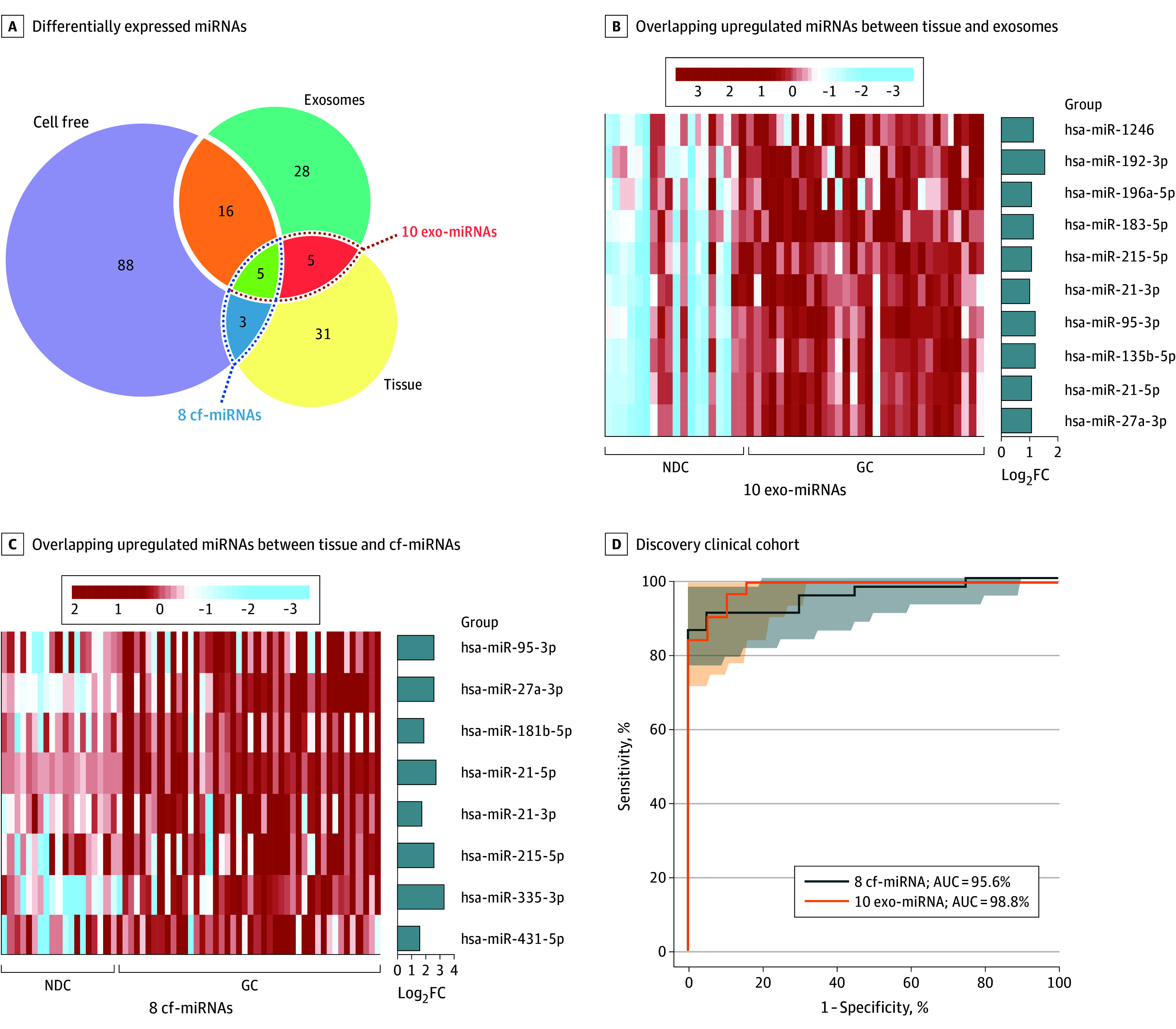
Genome-Wide Discovery of MicroRNA (miRNA) Candidates for Detection of Gastric Cancer A, Venn diagram shows the overlapping differentially expressed miRNAs among tissue, cell-free (cf), and exosomes (exo). The Vennerable package, version 3.1, in R software (R Project for Statistical Computing) was used to create the Venn diagram. B, The heatmap depicts the 10 overlapping upregulated miRNAs between tissue and exosomes (exo-miRNAs). C, The heatmap illustrates the 8 overlapping upregulated miRNAs between tissue and cell-free (cf-miRNAs). D, Receiver operating characteristic curve analysis evaluates the performance of 8 cf-miRNA panels and 10 exo-miRNA panels that robustly distinguished gastric cancer from controls without gastric cancer. ROC curves are shown as 95% CIs. AUC indicates area under the curve; FC, fold change; hsa, *Homo sapiens* (human miRNA).

To prioritize GC-specific biomarker candidates, we narrowed down a panel of 10 miRNAs (miR-21-3p, miR-21-5p, miR-1246, miR-192-3p, miR-215-5p, miR-27a-3p, miR-95-3p, miR-196a-5p, miR-183-5p, and miR-135b-5p) overlapping between tissue and exosomes (Figure 1A and B) and another panel of 8 miRNAs (miR-21-3p, miR-21-5p, miR-215-5p, miR-335-3p, miR-27a-3p, miR-95-3p, miR-181b-5p, and miR-431-5p) whose expression overlapped between tissue and cell-free fractions (Figure 1A and C). Interestingly, 5 miRNAs (miR-21-3p, miR-21-5p, miR-215-5p, miR-27a-3p, and miR-95-3p) were common to both cell-free and exosomal fractions (Figure 1A).

Subsequently, we performed logistic regression and calculated the risk scores within the 8 cell-free–miRNA and 10 exosomal-miRNA panels. ROC curve analysis revealed both panels were robust to identify patients with GC, with AUC values of 95.6% for the cell-free–miRNA panel and 98.8% for the exosomal-miRNA panel in the discovery cohort (Figure 1D). Collectively, our biomarker discovery efforts successfully developed unique cell-free–miRNA and exosomal-miRNA panels for early GC detection.

### Machine Learning–Based Training and Validation

To establish an miRNA biomarker panel for noninvasive GC diagnosis, we quantitated the expression levels of discovered miRNA candidates in serum samples from the training cohort (GC = 161 cases; controls without disease = 102 individuals) using reverse transcriptase quantitative polymerase chain reaction assays. One GC case was excluded due to undetectable expression levels. Consistent with sequence-profiling results, all 8 cell-free miRNAs and 9 of the 10 exosomal miRNAs (excluding miR-135-5p) exhibited significantly elevated expression in GC vs controls and were included in further analysis. The diagnostic performance of each cell-free– and exosomal-miRNA biomarker is summarized in eTables 2 and 3 in Supplement 1.

**Figure 2.**
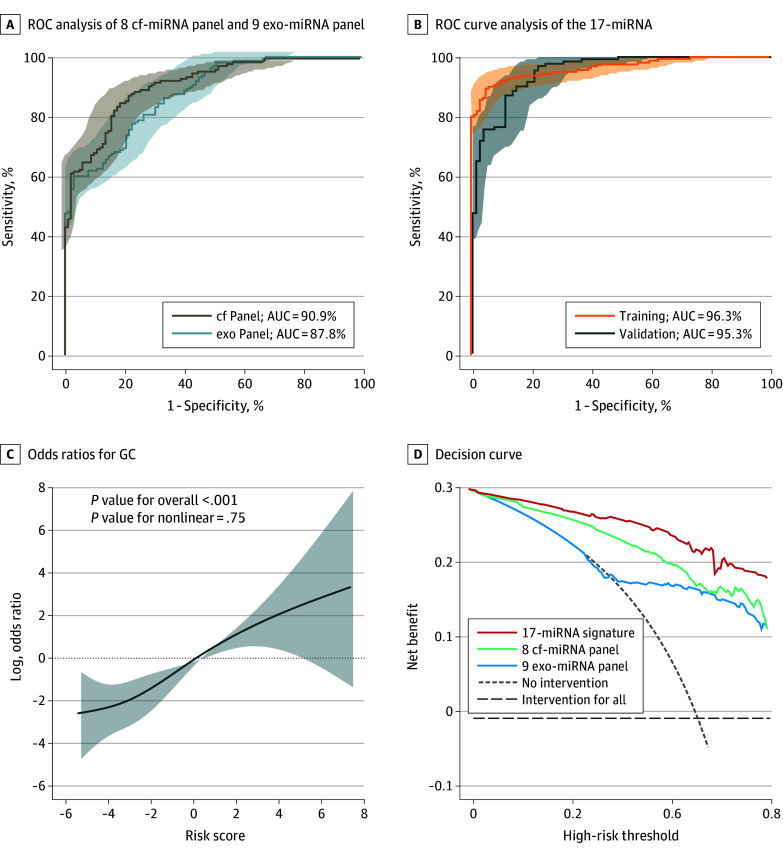
Development of a Noninvasive MicroRNA (miRNA)–Based Signature for Detection of Patients With Gastric Cancer (GC) A, Receiver operating characteristic (ROC) curve analysis evaluates the performance of 8 cell-free miRNA (cf-miRNA) panel and 9 exosomal miRNA (exo-miRNA) panel in the training cohort. ROC curves are shown as 95% CIs. B, ROC curve analysis illustrates the performance of the 17-miRNA combination signature in the training and validation cohort. ROC curves are shown as 95% CIs. C, Odds ratios for GC with restricted cubic splines as a function of risk score values (95% CIs for odds ratios are presented as dashed lines). D, The decision curve shows the net benefit for the cf-miRNA, exo-miRNA, and combination signature in patients with GC from the validation cohort. AUC indicates area under the curve.

Next, we applied an eXtreme Gradient Boosting (XGBoost)–based machine-learning algorithm (open source) to the training cohort. Separate panels of 8 cell-free miRNAs and 9 exosomal miRNAs were established, and risk scores were calculated using the XGBoost classifier. Density plots of cell-free–miRNA and exosomal-miRNA panels risk score revealed distinct clustering patterns, with controls without disease predominantly in the bottom left and patients with GC in the top right (eFigure 3A in Supplement 1). ROC analysis demonstrated remarkable performance for the 8 cell-free–miRNA panel (AUC = 90.9%; sensitivity = 85.0%; specificity = 81.4%) (Figure 2A) and the 9 exosomal-miRNA panel (AUC = 87.8%; sensitivity = 60.0%; specificity = 97.1%) (Figure 2A), highlighting their ability to accurately discriminate patients with GC from controls without disease. More importantly, the cell-free–miRNA panel exhibited higher sensitivity, and the exosomal-miRNA panel revealed greater specificity for GC detection.

Our hypothesis proposed that cell-free miRNAs may provide higher sensitivity, and exosomal miRNAs may provide greater specificity, with their combination offering optimal diagnostic accuracy. To test this, we combined markers from the 8 cell-free– and 9 exosomal-miRNA panels in the training cohort. Indeed, supporting our hypothesis, the diagnostic scores calculated based on the combined 17-miRNA signature were significantly higher in patients with GC compared with controls without disease (GC vs controls without disease: 4.02 vs −2.48; *P* <.001) (eFigure 3B in Supplement 1). Moreover, the 17-miRNA signature exhibited superior performance (AUC = 96.3%; 95% CI, 94.3%-98.4%;) (Figure 2B) vis-à-vis individual cell-free– and exosomal-miRNA panels (AUC = 90.9% and 87.8%, respectively). Waterfall plots further confirmed its effectiveness, identifying 89.4% true positives (143 of 160 cases) and 95.1% true negatives (97 of 102 individuals) (eFigure 3C in Supplement 1).

Next, we validated the diagnostic performance of the 17-miRNA signature in another independent validation cohort (GC = 131 cases; controls without disease = 86 individuals). Four individuals were excluded due to undetermined miRNA levels. In this serum-based validation phase, we applied the same XGBoost classifier model parameters from our locked-down assay in the training cohort to calculate risk scores. These markers performed consistently even in this cohort, with significantly higher diagnostic risk scores in patients with GC than in controls without disease (GC vs controls without disease: 2.89 vs −2.92; *P* <.001) (eFigure 3B in Supplement 1). ROC analysis revealed impressive performance for the 17-miRNA signature (AUC = 95.3%; 95% CI, 92.8%-97.9%) (Figure 2B), achieving 87.0% true positives (114 of 131 cases) and 89.0% true negatives (73 of 82 individuals) (eFigure 3C in Supplement 1). Interestingly, this signature exhibited a highly significant association with GC odd ratios (log odd ratio range, −2.88 to 2.63; SD = 1.83; *P* <.001), indicating a proportional increase in the risk of GC (Figure 2C).

Furthermore, we performed decision curve analysis (DCA) to determine the clinical utility of risk stratification for each biomarker panel in the validation cohort. The DCA curve demonstrated that the combined signature offered a significantly superior net benefit than the individual cell-free– and exosomal-miRNA panels, underscoring its potential to minimize harm and reduce misdiagnosis in clinical practice (Figure 2D). Collectively, these findings highlight that the 17-miRNA combination signature offers remarkable diagnostic ability for accurate identification of GC.

### Establishment of a Clinic-Friendly and Inexpensive miRNA Signature for Early Detection of GC

Interestingly, we observed a smaller subset of 5 miRNAs (miR-21-3p, miR-21-5p, miR-215-5p, miR-27a-3p, and miR-95-3p) that were shared between the cell-free– and exosomal-miRNA panels. To establish a clinically feasible and effective diagnostic assay with minimum number of biomarkers and optimal diagnostic performance, we compared the 10-miRNA signature (combination of 5 cell-free miRNA and 5 exosomal miRNA) with the 17-miRNA signature for GC identification. First, using an XGBoost algorithm, we established the 5 cell-free–miRNA and 5 exosomal-miRNA panels. Shapley additive explanations values highlighted the contributions of each miRNA within these panels (eFigure 4A in Supplement 1). Notably, the top 3 contributors were consistent across both panels (miR-21-3p, miR-21-5p, and miR-215-5p). The diagnostic ability of the 5-miRNA panel was slightly lower for the cell-free component (AUC = 5 cell-free–miRNAs vs 8 cell-free–miRNA panel: 87.0%; 95% CI, 82.7%-91.2% vs 90.9%; 95% CI, 87.6%-94.3%), but showed similar performance for exosomal markers (AUC = 5 exosomal-miRNA vs 8 exosomal-miRNA panel: 89.1%; 95% CI, 85.3%-92.8% vs 87.7%; 95% CI, 83.7%-91.6%). However, it was not apparent whether this reduced combination of 5 cell-free and exosomal miRNAs might be synergistic and would still result in a robust diagnostic assay with comparable or improved sensitivity and specificity vs the larger panels of markers mentioned earlier.

**Figure 3.**
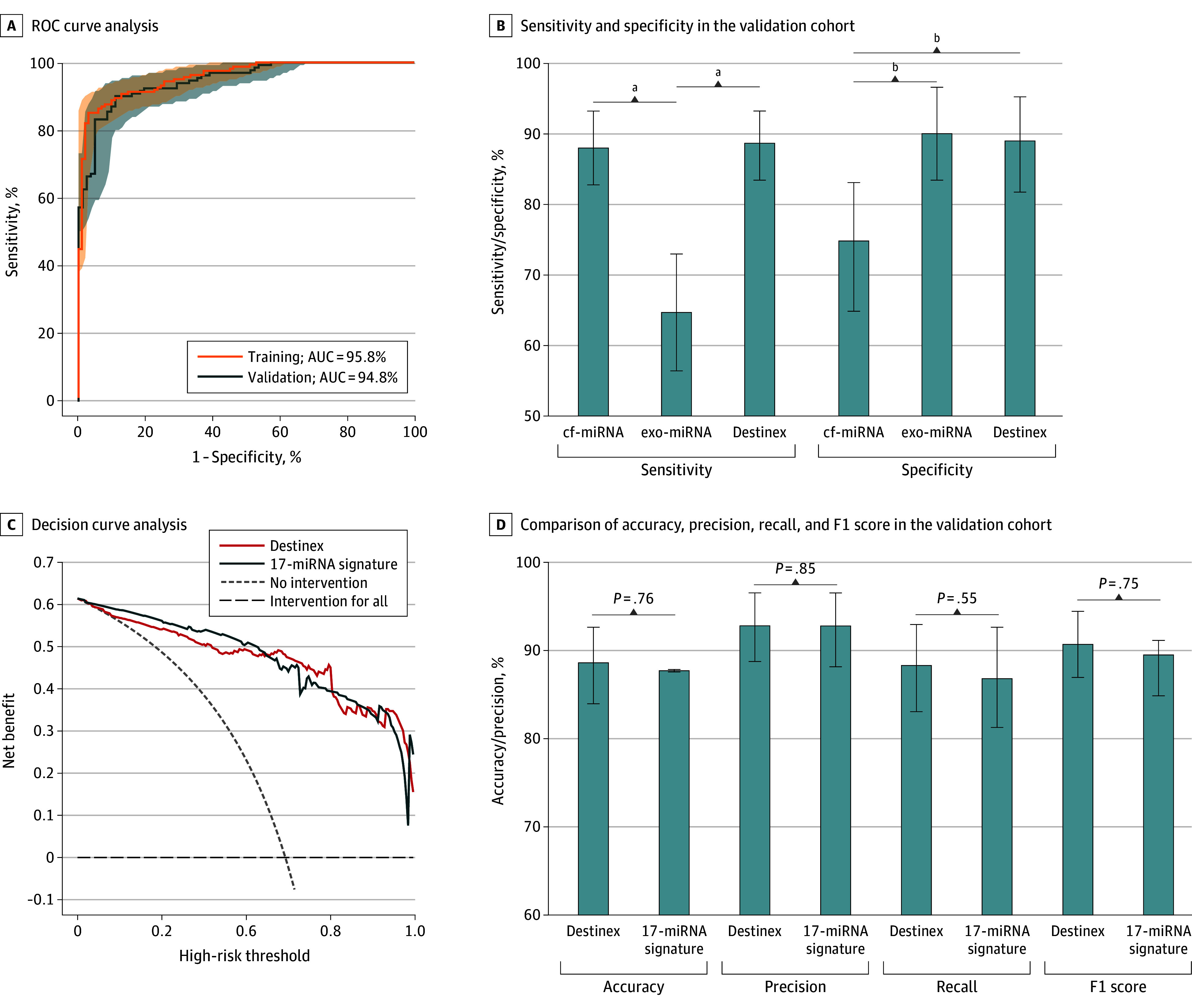
Construction of a Clinically Feasible Signature for Noninvasive Detection of Patients With Gastric Cancer (GC) A, Receiver operating characteristic (ROC) curve analysis illustrates the performance of the Destinex assay in the training and validation cohort. B, The sensitivity and specificity of 5 cell-free microRNA (cf-miRNA) panel, 5 exosomal miRNA (exo-miRNA) panel, and the Destinex assay were shown in the validation cohort. C, The decision curve analysis reveals the net benefit of the Destinex assay and 17-miRNA combination signatures in patients with GC from the validation cohort. The Destinex assay indicates detection of GC by combining 5 cf-miRNAs and exo-miRNAs. D, The comparison of accuracy, precision, recall, and F1 score between the Destinex assay and the 17-miRNA combination signature is shown in the validation cohort. AUC indicates area under the curve. ^a^*P* < .05. ^b^*P* < .001.

To test this hypothesis, we developed a final signature for the detection of gastric cancer by combining 5 cell-free and exosomal miRNAs (Destinex) and calculated risk probability for each patient in the training cohort. Destinex scores were significantly higher in patients with GC than in controls without disease (GC vs controls without disease: 4.30 vs −2.00; *P* <.001) (eFigure 4B in Supplement 1), with an AUC value of 95.8% (95% CI, 93.7%-97.9%) (Figure 3A). Given these results, the final Destinex assay was developed and fully locked, including all model parameters and expression threshold cutoffs. When applied to the validation cohort, the Destinex score remained significantly higher in patients with GC vs controls without disease (GC vs controls without disease: 3.80 vs -1.58; *P* < .001) (eFigure 4B in Supplement 1), achieving an AUC of 94.8% (95% CI, 92.1%-97.5%) (Figure 3A). Additionally, Destinex assay once again demonstrated higher sensitivity for cell-free miRNAs (5 cell-free vs 5 exosomal miRNAs = 87.8%; 95% CI, 82.4%-93.1% vs 64.9%; 95% CI, 57.2%-72.5%) and superior specificity for exosomal miRNAs (5 cell-free vs 5 exosomal miRNAs = 74.4%; 95% CI, 64.6%-82.9% vs 90.2%; 95% CI, 84.1%-93.6). More significantly, the Destinex assay, which harnesses the sensitivity and specificity of both cell-free and exosomal miRNAs, yielded an overall sensitivity of 88.5% and a specificity of 89.0% by using the same XGBoost classifier and cutoff (1.07) as the training cohort (Figure 3B).

Finally, we conducted a systematic comparative analysis of the diagnostic performance between Destinex and the original 17-miRNA signature. The AUC values, sensitivity, specificity, accuracy, positive predictive value (PPV), and negative predictive value (NPV) are summarized in the Table. An evaluation of accuracy, precision, recall, and F1 score in the validation cohorts indicated that Destinex remained consistently comparable with the 17-miRNA signature (Figure 3D). Moreover, DCA analysis for Destinex exhibited equally similar net benefit (Figure 3C). Similarly, calibration curve analysis reassured that the predicted probability of Destinex matched the actual probability of GC (eFigure 4C in Supplement 1) (Destinex: χ^2^ = 7.95; *P* = .63; 17-miRNA signature: χ^2^ = 5.9; *P* = .83). Collectively, Destinex robustly distinguished patients with GC from controls without disease, offering a powerful tool for effectively screening patients with GC.

**Table.** The Performance of the Destinex Assay and 17-MicroRNA (miRNA) Signature for Detection of Gastric Cancer (GC) in the Training and Validation Cohorts

Performance, %	Cohort			
	Training^a^		Validation^b^	
	Destinex @cutoff (1.065)	17-miRNA signature @cutoff (0.349)	Destinex @cutoff (1.065)	17-miRNA signature @cutoff (0.349)
AUC (95% CI)	95.8 (93.7-97.9)	96.3 (94.3-98.4)	94.8 (92.1-97.5)	95.3 (92.8-97.9)
Sensitivity (95% CI)	85.0 (79.5-90.5)	89.4 (84.6-94.1)	88.5 (83.2-93.1)	70.2 (61.8-77.9)
Specificity (95% CI)	97.1 (93.8-100.0)	95.1 (90.9-99.3)	89.0 (81.7-95.1)	97.6 (93.9-100.0)
Accuracy (95% CI)	89.7 (89.6-89.8)	91.6 (91.5-91.7)	88.7 (84.0-93.0)	80.8 (75.6-58.9)
PPV (95% CI)	97.8 (95.4-100.0)	96.6 (93.7-99.5)	92.9 (88.8-96.7)	97.9 (95.0-100.0)
NPV (95% CI)	80.5 (73.5-87.5)	85.1 (78.5-91.6)	83.1 (76.1-89.5)	67.1 (61.7-73.4)

Abbreviations: AUC, area under the curve; NPV, negative predictive value; PPV, positive predictive value.

^a^ Based on the Youden cutoff value.

^b^ The cutoff value was determined from the training cohort.

### Noninvasive miRNA Signature and Patients With Early-Stage GC

**Figure 4.**
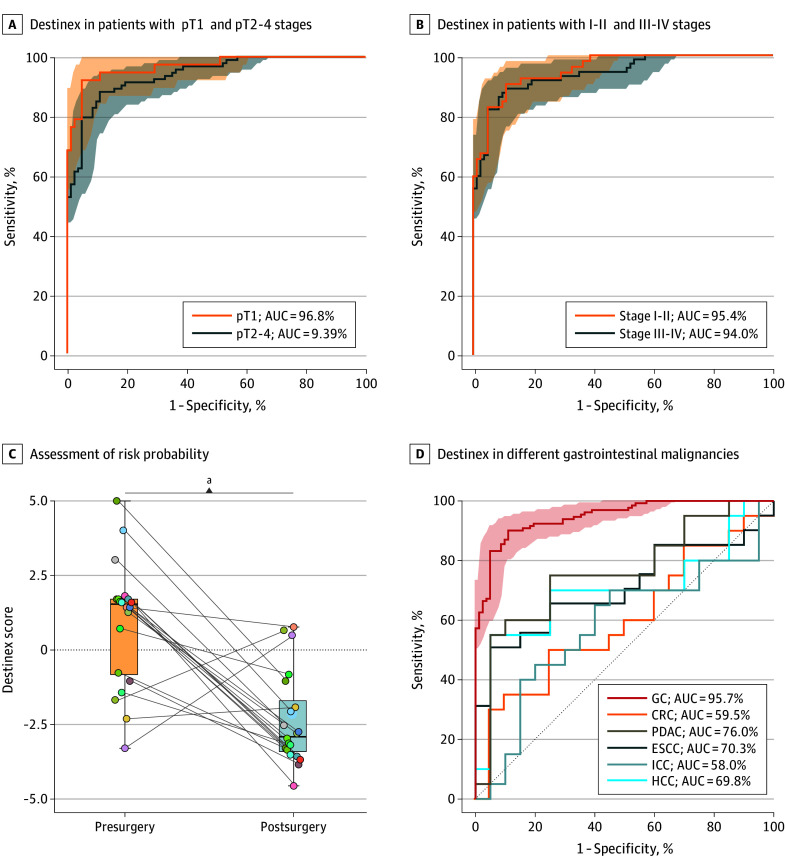
The Noninvasive Destinex Assay Efficiently Identifies Patients With Early Gastric Cancer (GC) A, Receiver operating characteristic (ROC) curve analysis reveals the performance of the Destinex assay in patients with the pathological tumor stage 1 (pT1) and pT2-4 stages from the validation cohort. B, ROC curve analysis reveals the performance of the Destinex assay in patients with stages I-II and III-IV from the validation cohort. ROC curves are shown as 95% CIs. C, Assessment of risk probability based on the Destinex assay between presurgery and postsurgery GC samples. D, ROC curve analysis shows the performance of the Destinex assay in different gastrointestinal malignancies (GC, colorectal cancer [CRC], pancreatic ductal adenocarcinoma cancer [PDAC], esophageal squamous cell carcinoma [ESCC], intrahepatic cholangiocarcinoma [ICC], hepatocellular carcinoma [HCC]). The Destinex assay indicates detection of GC by combining 5 cell-free microRNAs (cf-miRNAs) and exosomal miRNAs (exo-miRNAs). AUC indicates area under the curve. ^a^*P* < .001.

Early diagnosis is a necessary health care strategy in all settings, as it significantly improves clinical outcomes. Early GC is defined as cancer confined to the mucosal or submucosal layer (stage T1), regardless of lymph node metastasis. Importantly, our results revealed that Destinex risk scores were consistently higher even in patients with pT1 stage (pT1 vs pT2-4 vs controls without disease = 3.92 vs 3.76 vs −1.58; *P* < .001) (eFigure 5A in Supplement 1). The Destinex assay exhibited superior ability in detecting early GC in the validation cohort (AUC of pT1 vs pT2-4: 96.8%; 95% CI, 93.5%-100% vs 93.9%; 95% CI, 90.7%-97.2%) (Figure 4A). Additionally, the Destinex scores were also significantly higher in patients with GC, across all stages from I to IV (I-II vs III-IV vs controls without disease = 3.90 vs 3.71 vs −1.58; *P* < .001) (eFigure 5B in Supplement 1). ROC analysis further highlighted Destinex’s robust identification of early GC (stage I and II: AUC = 95.4%; 95% CI, 92.2%-98.5%; stage III and IV: AUC = 94.0%; 95% CI, 90.4%-97.6%) (Figure 4B).

Next, we evaluated the sensitivity of Destinex in various subsets of patients with GC based on clinicopathological features (pT stage, TNM [tumor-node-metastasis] stage, pathology, lymphatic invasion) (eFigure 5C in Supplement 1). Using the locked cutoff identified from the training cohort, Destinex exhibited significantly higher sensitivity for detecting pT1 stage cancers (pT1 vs pT2-4: 95% vs 86%) and for patients with positive lymph node invasion (yes vs no: 89% vs 85%). Additionally, to assess the diagnostic performance of Destinex in identifying early- and late-stage GC, we conducted χ^2^ tests comparing sensitivity and AUC between different stages. The results demonstrated that Destinex assay maintained similarly robust performance across early- and late-stage GC.

We further investigated the diagnostic performance of Destinex regarding their specificity at various sensitivity thresholds of 90%, 92.5%, and 95% for early GC detection in the validation cohort, summarized in eTable 4 in Supplement 1. The results revealed that Destinex had a remarkable sensitivity even at a specificity of 90% (pT1 vs pT2-4: 93.9% vs 82.9%) for the detection of patients with early GC. Taken together, Destinex could robustly distinguish patients with early-stage GC from controls without disease.

### Destinex Assay Performance in Postsurgical Blood Samples and Other GCs

To further investigate Destinex specificity for GC detection, we first analyzed circulating cell-free– and exosomal-miRNA levels (miR-21-3p, miR-21-5p, miR-215-5p, miR-27a-3p, miR-95-3p) in serum samples from a subset of 20 patients with GC (eTable 5 in Supplement 1), comparing presurgery blood samples with matched specimens collected 3 months postsurgery. We hypothesized that if the elevated cell-free– and exosomal-miRNA levels in Destinex were tumor derived, their expression would decrease after tumor removal. As expected, other than exosomal miR-95-3p, the expression of all cell-free and exosomal miRNAs was significantly decreased in after surgery blood serum specimens (eFigure 6 in Supplement 1). Additionally, Destinex risk probabilities were significantly reduced in postoperative serum specimens (presurgery vs postsurgery = 0.90 vs −2.37; *P* < .001) (Figure 4C), highlighting that these markers are highly correlated with the presence of gastric neoplasia.

To ensure the specificity of Destinex for GC, we compared its diagnostic performance in patients with several other gastrointestinal cancers, including colorectal cancer (CRC), pancreatic ductal adenocarcinoma (PDAC), esophageal squamous cell carcinoma (ESCC), intrahepatic cholangiocarcinoma (ICC), and hepatocellular carcinoma (HCC). It was intriguing to witness that Destinex exhibited the highest diagnostic value for GC vs all other gastrointestinal cancers (Figure 4D) (GC: AUC = 95.7%; 95% CI, 93.7%-97.9%; CRC: AUC = 59.5%; 95% CI, 41.3%-77.7%; PDAC: AUC = 76.0%; 95% CI, 60.4%-91.6%; ESCC: AUC = 70.3%; 95% CI, 53.1%-87.4%; ICC: AUC = 58.0%; 95% CI, 39.3%-76.7%; HCC: AUC = 69.8%; 95% CI, 52.2%-87.3%), all with statistically significant differences.

Finally, to further elucidate the biological relevance of the identified miRNAs, we first predicted the target mRNAs of miR-21-3p, miR-21-5p, miR-215-5p, miR-27a-3p, and miR-95-3p using 3 databases: TargetScan, miRTarBase, and miRDB. We subsequently constructed an miRNA-mRNA regulatory network based on target genes that were consistently identified by at least 2 of these databases. Notably, several target genes—including *WNK1*, *CDK6*, *NCOA3*, and *NFAT5*—are functionally linked to key oncogenic processes such as tumor growth, proliferation, and metastasis (eFigure 7A in Supplement 1). Moreover, the core miRNAs included in the Destinex assay play pivotal roles in cancer-related pathways, particularly in regulating pluripotency of stem cells, FoxO signaling pathway, and the ErbB pathway (eFigure 7B in Supplement 1). Altogether, these results suggest that the noninvasive diagnostic Destinex signature was highly specific in detecting GC, which might have tremendous potential for routine clinical use in the future.

## Discussion

Despite declining global incidence, GC remains the third leading cause of cancer deaths, largely due to late-stage diagnosis, especially in countries without screening programs.^24^ Endoscopic screening, common in high-incidence regions, faces challenges such as patient discomfort, poor compliance, and high costs,^25^ emphasizing the need for noninvasive and affordable diagnostic methods for early GC detection.

Currently, primary analytes for liquid biopsy include CTCs, ctDNA, and extracellular vesicles, including exosomes. CTCs are challenging as biomarkers due to their rarity and heterogeneity,^26^ and ctDNA, although specific, lacks adequate sensitivity for early cancer detection.^27^ Conversely, although cell-free RNAs are potential biomarkers for several cancers including GC,^28,29^ they may lack specificity as they are released from various cellular sources. Extracellular vesicles are increasingly recognized as potential biomarkers due to their abundance in the bloodstream and the stability of their encapsulated content.^18,30^ Exosomal miRNAs, in particular, are attractive for noninvasive tumor diagnosis as they can identify cell types.^31,32^ This study suggested that cell-free miRNAs offer higher sensitivity, whereas exosomal miRNAs offer higher specificity to identify GC—a concept previously shown by our team in pancreatic cancer.^23^ Notably, the 17-miRNA signature exhibited a superior ability for accurate GC identification vs the cell-free–miRNA or exosomal-miRNA panels individually. Finally, we developed a signature, Destinex, comprising 5 overlapping cell-free and exosomal miRNAs, which yielded an impressive diagnostic performance with an AUC of 94.8% in the validation cohort. These results highlight that cell-free panel and exosomal panel could complement each other in enhancing sensitivity and specificity, resulting in improved diagnostic accuracy. More importantly, Destinex showed higher sensitivity in distinguishing early GC from controls without disease (pT1 vs pT2-4: 94.7% vs 86.0%), highlighting that Destinex is equally effective, more feasible and economical for detecting patients with early GC compared with the 17-miRNA signature.

The tumor-specific origin of these circulating biomarkers was confirmed by significantly decreased expression levels in postsurgical serum specimens. Previous studies have shown that the 5 miRNA biomarkers in our risk stratification signature, including miR-21-3p,^9,33,34^ miR-21-5p,^35,36,37^ miR-215-5p,^38,39^ miR-27a-3p,^40,41^ miR-95-3p^42^ were aberrantly expressed in various tumors, including GC. Functionally, several studies have demonstrated that miR-21 plays a central role in tumorigenesis, progression, metastasis, and its overexpression is related to poor survival in gastrointestinal cancers.^9,43,44^ Although some studies suggest that miR-215-5p may suppress epithelial-mesenchymal transition and inhibit tumor invasion and metastasis,^45^ compelling evidence indicates that it serves as a sensitive biomarker for early tumorigenesis, particularly in circulation and exosome-derived compartments.^38,46^ miR-27a-3p has been found to promote tumorigenesis and facilitate metastasis in gastrointestinal malignancies, including GC.^47,48^ miR-95-3p promotes chemotherapy resistance in human gastric cancer cells by targeting the PI3K/AKT pathway.^49^ These findings gave evidence of the tremendous potential of miRNA biomarkers to detect patients with GC.

### Limitations

Although promising, our findings have potential limitations. First, age and sex were unevenly distributed among those with GC and controls without disease. Second, we did not enroll patients with chronic atrophic gastritis and intestinal metaplasia. Although such biospecimen cohorts were not available to us at the time, we remain highly interested in evaluating these lesions in the near future. Third, Destinex showed superior accuracy for GC, although further validation is needed due to the limited sample size of other cancers. Finally, our serum-based validation was limited to Asian cohorts, highlighting the need for a multinational study to further validate the diagnostic efficiency of miRNA signatures before clinical application.

## Conclusions

In this case-control study, we identified and established a Destinex assay for the early and noninvasive detection of GC using a comprehensive biomarker discovery effort, followed by machine learning–based clinical training and validation in 2 independent cohorts of patients with GC and controls without disease. Our findings suggest the clinical significance of Destinex as a robust, noninvasive, potential assay for the early identification of GC.

## Notes

Supplement 1.
eMethods.
**eFigure 1.** Workflow of the Study Design for Developing a Noninvasive miRNA Panel for the Detection of GC **eFigure 2.** Differential Expression of Tissue, Exosomal, and Cell-Free miRNAs in GC **eFigure 3.** Construction of a Noninvasive miRNA-Based Signature for the Identification of Patients With GC **eFigure 4.** Construction of a Clinically Feasible Signature for Noninvasive Detection of Patients With GC **eFigure 5.** The Noninvasive DESTINEX Efficiently Identifies Patients With Early GC **eFigure 6.** The 10 Individual miRNAs Exhibited Robust Specificity for Detecting GC **eFigure 7.** Functional Analysis of miRNA-Target Interactions and Enriched Pathways **eTable 1.** Clinicopathological Characteristics of Enrolled Patients in the Training and Validation Cohorts **eTable 2.** Summary of the Diagnostic Performance of Individual Cell-Free miRNAs in the Training Cohort **eTable 3.** Summary of the Diagnostic Performance of Individual Exosomal miRNAs in the Training Cohort **eTable 4.** The Diagnostic Performance of DESTINEX at Varying Sensitivity Thresholds for Detection of pT1 GC **eTable 5.** Clinicopathological Characteristics of the 20 Patients With Paired Before and After Surgery Blood Samples

Supplement 2.
Data Sharing Statement.
