# Supplementary material for: Exosomal Liquid Biopsy for the Early Detection of Gastric Cancer: The DESTINEX Multicenter Study
Source: JAMA Surg. 2025 Jul 30;160(9):973–82. doi: 10.1001/jamasurg.2025.2493 (PMC12311825; doi:10.1001/jamasurg.2025.2493)
Supplement: Supplement 1. — eMethods. eFigure 1. Workflow of the Study Design for Developing a Noninvasive miRNA Panel for the Detection of GC eFigure 2. Differential Expression of Tissue, Exosomal, and Cell-Free miRNAs in GC eFigure 3. Construction of a Noninvasive miRNA-Based Signature for the Identification of Patients With GC eFigure 4. Construction of a Clinically Feasible Signature for Noninvasive Detection of Patients With GC eFigure 5. The Noninvasive DESTINEX Efficiently Identifies Patients With Early GC eFigure 6. The 10 Individual miRNAs Exhibited Robust Specificity for Detecting GC eFigure 7. Functional Analysis of miRNA-Target Interactions and Enriched Pathways eTable 1. Clinicopathological Characteristics of Enrolled Patients in the Training and Validation Cohorts eTable 2. Summary of the Diagnostic Performance of Individual Cell-Free miRNAs in the Training Cohort eTable 3. Summary of the Diagnostic Performance of Individual Exosomal miRNAs in the Training Cohort eTable 4. The Diagnostic Performance of DESTINEX at Varying Sensitivity Thresholds for Detection of pT1 GC eTable 5. Clinicopathological Characteristics of the 20 Patients With Paired Before and After Surgery Blood Samples [file jamasurg-e252493-s001.pdf]

Supplementary Online Content

Sui S, Xu C, Kanda M, et al. Exosomal liquid biopsy for the early detection of gastric cancer: the DESTINEX multicenter study. *JAMA Surg*. Published online July 30, 2025. doi:10.1001/jamasurg.2025.2493

eMethods.

eFigure 1. Workflow of the Study Design for Developing a Noninvasive miRNA Panel for the Detection of GC

eFigure 2. Differential Expression of Tissue, Exosomal, and Cell-Free miRNAs in GC

eFigure 3. Construction of a Noninvasive miRNA-Based Signature for the Identification of Patients With GC

eFigure 4. Construction of a Clinically Feasible Signature for Noninvasive Detection of Patients With GC

eFigure 5. The Noninvasive DESTINEX Efficiently Identifies Patients With Early GC

eFigure 6. The 10 Individual miRNAs Exhibited Robust Specificity for Detecting GC

eFigure 7. Functional Analysis of miRNA-Target Interactions and Enriched Pathways

eTable 1. Clinicopathological Characteristics of Enrolled Patients in the Training and Validation Cohorts

eTable 2. Summary of the Diagnostic Performance of Individual Cell-Free miRNAs in the Training Cohort

eTable 3. Summary of the Diagnostic Performance of Individual Exosomal miRNAs in the Training Cohort

eTable 4. The Diagnostic Performance of DESTINEX at Varying Sensitivity Thresholds for Detection of pT1 GC

eTable 5. Clinicopathological Characteristics of the 20 Patients With Paired Before and After Surgery Blood Samples

This supplementary material has been provided by the authors to give readers additional information about their work.

© 2025 Sui S et al. *JAMA Surgery*.

eMethods

Study design and patient cohorts.

For the biomarker discovery phase, we leveraged the expertise of multiple institutions. We performed small-RNA sequencing (small-RNA-Seq) using RNA samples from 47 GC tissues and matched AN tissues, cell-free RNA (cf-RNA), and exosomal RNA (exo-RNA) from 20 NDCs (Asan, South Korea), and cf- and exo-RNA from 43 and 32 GC patients, respectively from Ajou University (Suwon, South Korea). This collaborative effort allowed us to analyze the sequencing data for biomarker discovery using elaborate bioinformatic approaches to identify and prioritize biomarkers for the subsequent training phase. For the serum-based training phase, we performed real-time quantitative reverse transcription polymerase chain reaction (RT-qPCR) assays to evaluate the expression levels of cf- and exo-miRNAs in 263 serum specimens collected from 161 GC patients and 102 NDCs who were enrolled at the Nagoya University Hospital (Nagoya, Japan) between 2016 and 2020. For the serum-based validation phase, we investigated the performance of the trained cf- and exo-miRNAs panel in an additional independent clinical cohort comprised of 131 GC patients enrolled at Ajou University (Suwon, Korea) between 2017 and 2020 and 86 NDC subjects enrolled at Asan Medical Center (Seoul, Korea) and Samsung Medical Center (Seoul, Korea) between 2009 and 2017. To evaluate the diagnostic performance of the final validated miRNA signature, 20 matched pairs of pre- and post-operative serum specimens from patients with GC enrolled at Ajou University (Suwon, Korea) were analyzed. Finally, to confirm the specificity of our combination signature for GC, we compared its performance with other gastrointestinal cancers by analyzing serum specimens from 20 cases each of ESCC, PDAC from patients enrolled at Nagoya University (Nagoya, Japan), CRC cases from the Mie University (Mie, Japan), and HCC and ICC cases from Hokkaido University (Hokkaido, Japan).

All participants provided written informed consent. The study was approved by the IRBs of all participating institutions—including Asan Medical Center, Ajou University, Samsung Medical Center, Nagoya University, Hokkaido University, Mie University, and City of Hope—and conducted in accordance with the Declaration of Helsinki. The protocol was registered on ClinicalTrials.gov (NCT06342427).

Definitions, inclusion and exclusion criteria, and study endpoints

Eligible cases were defined as individuals with a histological diagnosis of GC according to the 8th edition of the AJCC Staging System. NDCs were defined as individuals without GC at the time of blood collection and with at least one year of confirmed negative follow-up. Blood samples for training and validation were collected before, during, and after treatment to evaluate the specificity of the test. The primary exclusion criterion for cases was a history of other malignancies. The primary outcome of this study was to determine the sensitivity for detecting patients with GC. Secondary outcome measures included sensitivity for early-stage (pT1) GC. Exploratory analyses evaluated the test's sensitivity for GC detection across various anatomical sites, ethnic groups, and geographic regions, as well as its specificity during treatment.

Tissue, cell-free, and exosomal RNA extraction

Tissue samples were collected from patients with GC, immediately preserved in RNAlater after surgical excision, and stored at -80°C. Blood samples were obtained prior to treatment, centrifuged at 3000 g for ten minutes within 12 hours after collection, and stored at -80°C.

To prepare small RNA sequencing libraries from the tissue specimens, total RNA was isolated from fresh-frozen GC tissues and matched AN tissues using AllPrep DNA/RNA/miRNA Universal Kit (Qiagen, Valencia, CA, USA). Similarly, to generate the small RNA sequencing libraries for blood specimens, the total cf- and exo-RNA were isolated from 400 µl serum using the miRNeasy kit and exoRNeasy Midi Kit (Qiagen), respectively. For the qRT-

© 2025 Sui S et al. *JAMA Surgery.*

PCR assays, exosomes were initially isolated from 200 μl serum using a Total Exosome Isolation Kit (Invitrogen, Waltham, MA, USA) and RNA extraction using the miRNeasy Kit (Qiagen). Cell-free total RNA was isolated from 200 μl serum using the miRNeasy Kit directly (Qiagen).

Small RNA sequencing

Total tissue RNA, cf-RNA, and exo-RNA were used for generating small-RNA-Seq libraries using NEXTflex Small RNA-Seq Kit v3 (PerkinElmer, Waltham, MA). After size exclusion and quality assurance, the sequencing libraries were pooled, and paired-end sequencing was performed on an Illumina NovaSeq platform. For the analysis of raw sequencing data, following quality control by FASTQC (v0.12) and adaptor trimming by Cutadapt (v3.4), the miRDeep2 tool was used for miRNA alignment (against miRbase release 22) and quantification of miRNA expression.

Biomarker discovery analysis for identification of cf- and exo-miRNAs

We analyzed genome-wide small-RNA-Seq expression profiling data during the biomarker discovery phase to identify differentially expressed miRNAs (DEMs) in patients with GC. The differentially expressed gene analysis was performed using the 'limma' package[1]. The tissue DEMs were filtered at a $|\log_2$foldchange ($\log_2$FC) $| > 0.5$ and a p-value < 0.01. Cf- and exo-miRNAs were filtered at a $\log_2$FC > 1 and a p-value < 0.01. Finally, the miRNA candidates that overlapped between tissue, cell-free, and exosomal fractions were selected for further biomarker training and validation phases.

RT-qPCR assays

The complementary DNA (cDNA) was synthesized using the miRCURY LNA RT Kit (Qiagen). The expression level of each miRNA candidate was quantified using a SensiFAST™ SYBR® LO-ROX Kit (Bioline, London, UK) on a QuantStudio 7 flex real-time quantitative PCR system (Applied Biosystems, Foster City, CA). RNU6B and miR-16-5p were determined to be the optimal controls in cf- and exo-RNA, respectively, based on their stable expression across all samples (GC and NDC). The expression values of miRNAs were calculated by the $2^{-\Delta CT}$ method.

Model Architecture and Hyperparameters

Our final liquid biopsy diagnostic assay, named *DESTINEX*, was trained by XGBoost (eXtreme Gradient Boosting). XGBoost is an optimized gradient tree boosting system incorporating algorithmic innovations like approximate greedy search, parallel learning, and various hyperparameters to enhance learning and control overfitting. XGBoost has been successfully applied to many studies [2,3]. In this study, the final XGBoost model was trained using gradient boosting trees by the 'xgboost' package in R (Version 0.1.3). We employed the AUC/precision-recall ('aucpr') evaluation metric to train a binary discriminatory assay to optimize the accuracy. The final locked model had the following parameters and hyperparameters: nrounds = 100, max_depth = 4, colsample_bytree = 1, eta = 0.01, verbosity = 1, min_child_weight = 1, scale_pos_weight = 1. We assessed feature importance using the "gain" metric calculated by XGBoost, which indicates the relative contribution of each of the final included miRNAs to the accuracy of the model. The model was developed, fully trained on the training cohort, and applied to the validation cohort.

Statistical analysis

All statistical analyses were performed using R and GraphPad Prism. The differential expression analysis was performed using the "limma" package in R. To evaluate the performance of diagnostic biomarkers, the receiver operating characteristic curve (ROC) analysis was conducted using the "pROC" package. The area under the receiver operating characteristic curves (AUCs) with 95% confidence intervals (CI) was computed by the method

of DeLong, with optimal cutoff thresholds determined by Youden's index. The decision curve analysis (DCA) was developed to delineate the net benefit value of the miRNA signature by using the "rmda" function. Calibration curve analysis was applied to assess the calibration of the miRNA signature using the "CalibrationCurves" function in R. The Mann-Whitney U and t-test were applied to compare two independent groups with continuous variables. Additionally, the chi-square test was used to compare AUC and sensitivity across different subgroups. A *p*-value of < 0.05 was considered a statistically significant change.

REFERENCES

1. Ritchie ME, Phipson B, Wu D, et al. limma powers differential expression analyses for RNA-sequencing and microarray studies. *Nucleic Acids Res*. Apr 20 2015;43(7):e47. doi:10.1093/nar/gkv007
2. Mukherjee S, Patra A, Khasawneh H, et al. Radiomics-based Machine-learning Models Can Detect Pancreatic Cancer on Prediagnostic Computed Tomography Scans at a Substantial Lead Time Before Clinical Diagnosis. *Gastroenterology*. Nov 2022;163(5):1435-1446 e3. doi:10.1053/j.gastro.2022.06.066
3. Khera R, Haimovich J, Hurley NC, et al. Use of Machine Learning Models to Predict Death After Acute Myocardial Infarction. *JAMA Cardiol*. Jun 1 2021;6(6):633-641. doi:10.1001/jamacardio.2021.0122

© 2025 Sui S et al. *JAMA Surgery*.

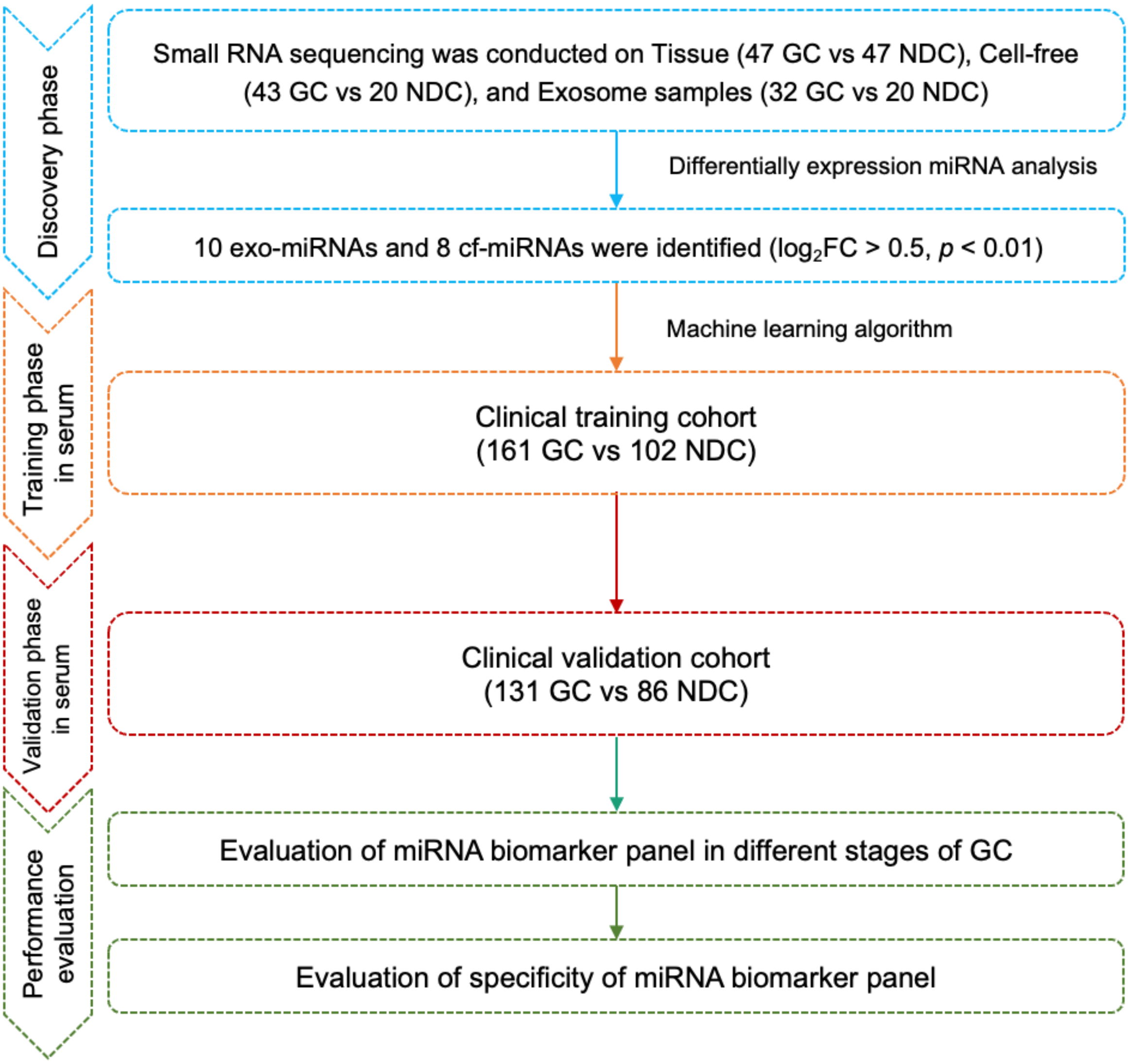

eFigure 1. Workflow of the study design for developing a non-invasive miRNA panel for the detection of GC. GC: Gastric cancer; NDCs: Non-disease controls; FC: Fold change

© 2025 Sui S et al. *JAMA Surgery*.

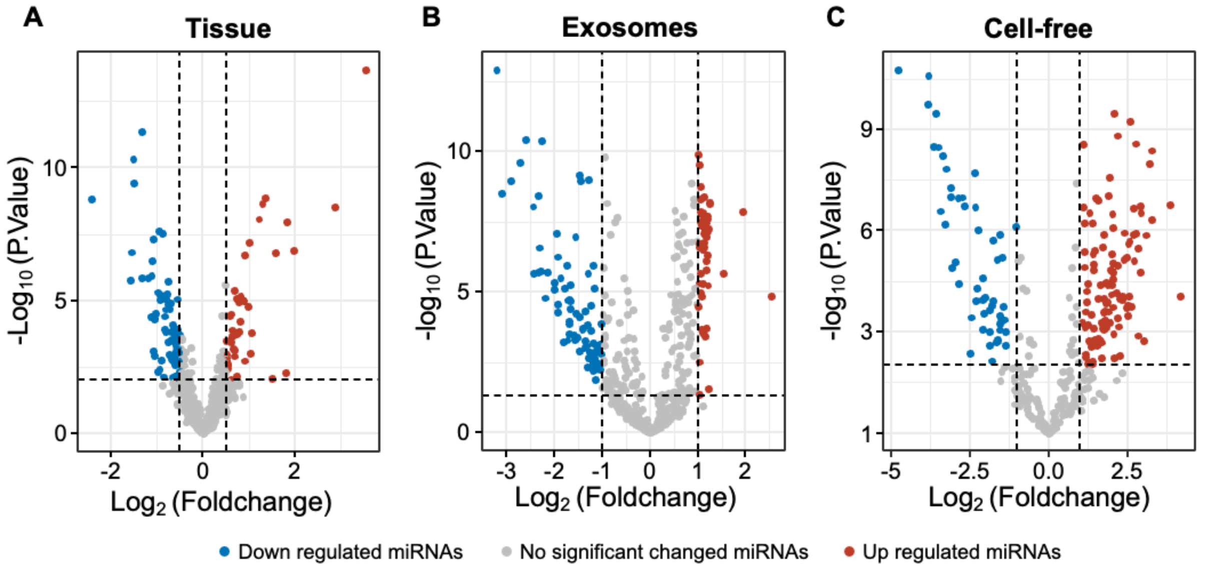

eFigure 2. Differential Expression of Tissue, Exosomal, and Cell-free miRNAs in GC.

A. Volcano plots depict differentially expressed miRNAs (DEMs) between gastric cancer (GC) tissue and matched adjacent normal (AN) tissues (|$\log_2$foldchange ($\log_2$FC)| > 0.5 and p < 0.01). **B.** Volcano plots depict differentially expressed exosomal (exo) miRNAs between GC and NDCs ($\log_2$FC > 1 and p < 0.01). **C.** Volcano plots depict differentially expressed cell-free (cf) miRNAs between GC and non-disease controls (NDCs) ($\log_2$FC > 1 and p < 0.01).

© 2025 Sui S et al. *JAMA Surgery.*

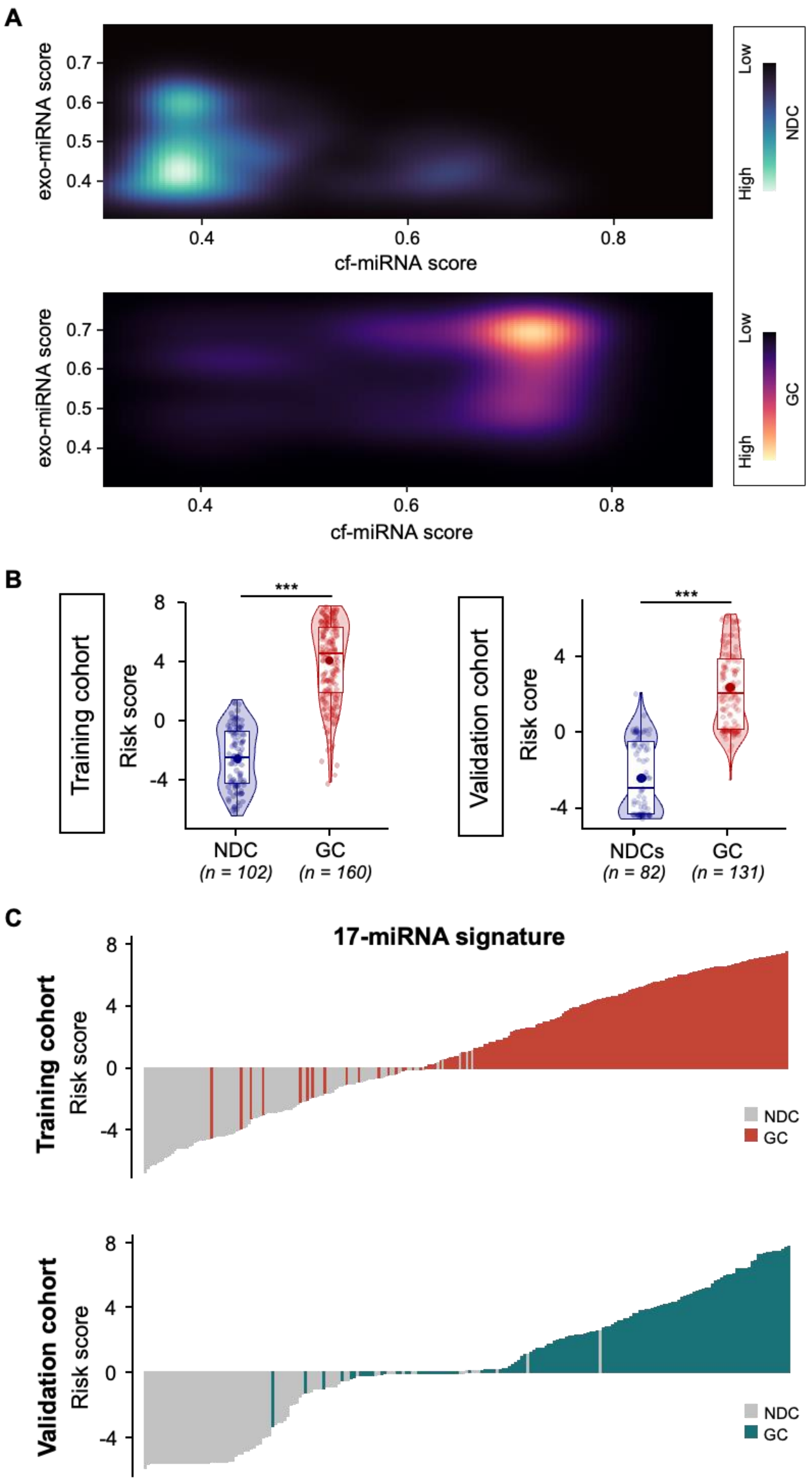

© 2025 Sui S et al. *JAMA Surgery*.

eFigure 3. Construction of a non-invasive miRNA-based signature for the identification of patients with GC. **A.** The density plots of the cf- and exo-miRNA panels for gastric cancer (GC; red) and non-disease controls (NDCs; blue) show distinct separation between the two groups. GC cases cluster in the top-right corner, while controls cluster in the bottom-left. **B.** The risk score calculated from the 17-miRNA signature, based on the machine learning model, was shown in the training and validation cohort. **C.** The waterfall plot exhibits the risk probability distribution of the combined 17-miRNA signature between serum samples from GC patients and NDCs in training and validation cohort. *** $p < 0.001$

© 2025 Sui S et al. *JAMA Surgery.*

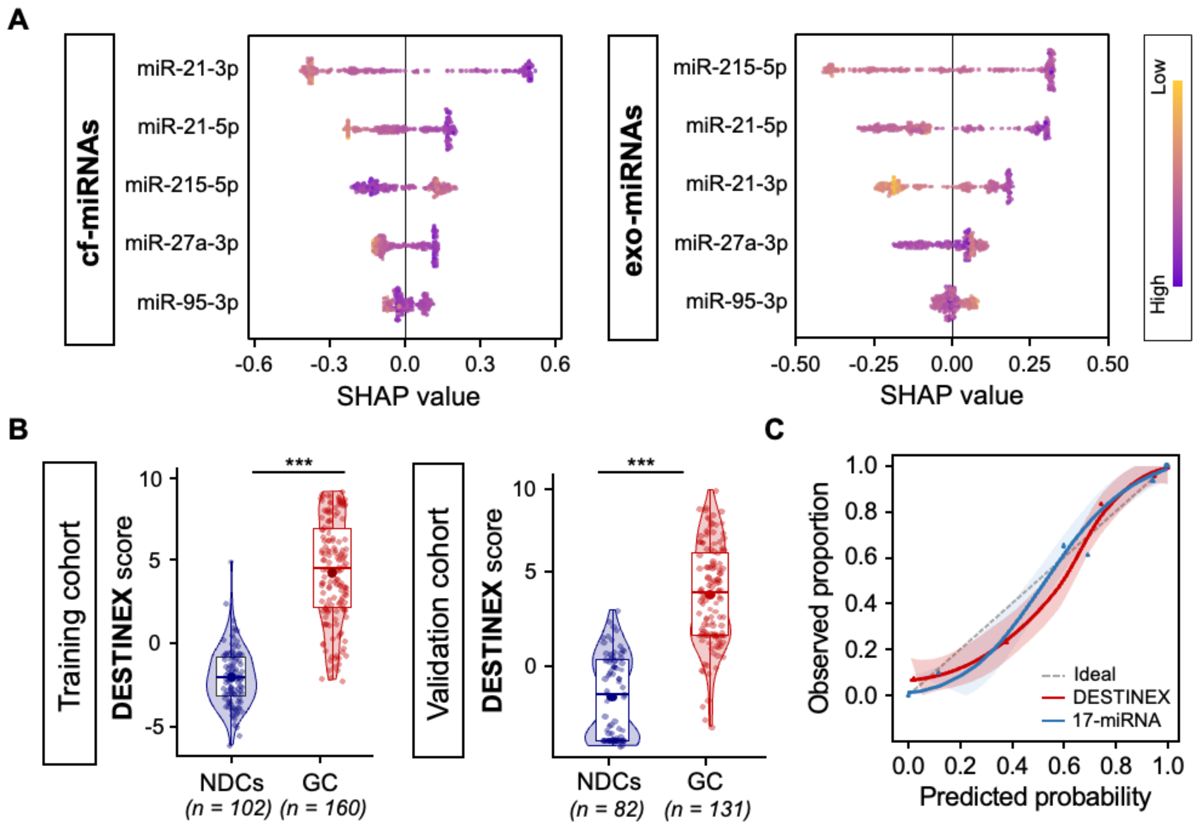

eFigure 4. Construction of a clinically feasible signature for non-invasive detection of patients with GC. **A.** SHAP plot illustrates the feature importance ranked in descending order on the Y-axis, with the SHAP values represented on the X-axis. **B.** The risk score calculated from DESTINEX was shown in the training and validation cohort. **C.** Calibration curves for DESTINEX and 17-miRNA signature in patients with GC from validation cohort. The dashed grey line is the ideal line. The triangle sign indicates the grouped observations. The short line above and below the horizontal axis represents the positive and negative cases. *** $p < 0.001$

© 2025 Sui S et al. *JAMA Surgery*.

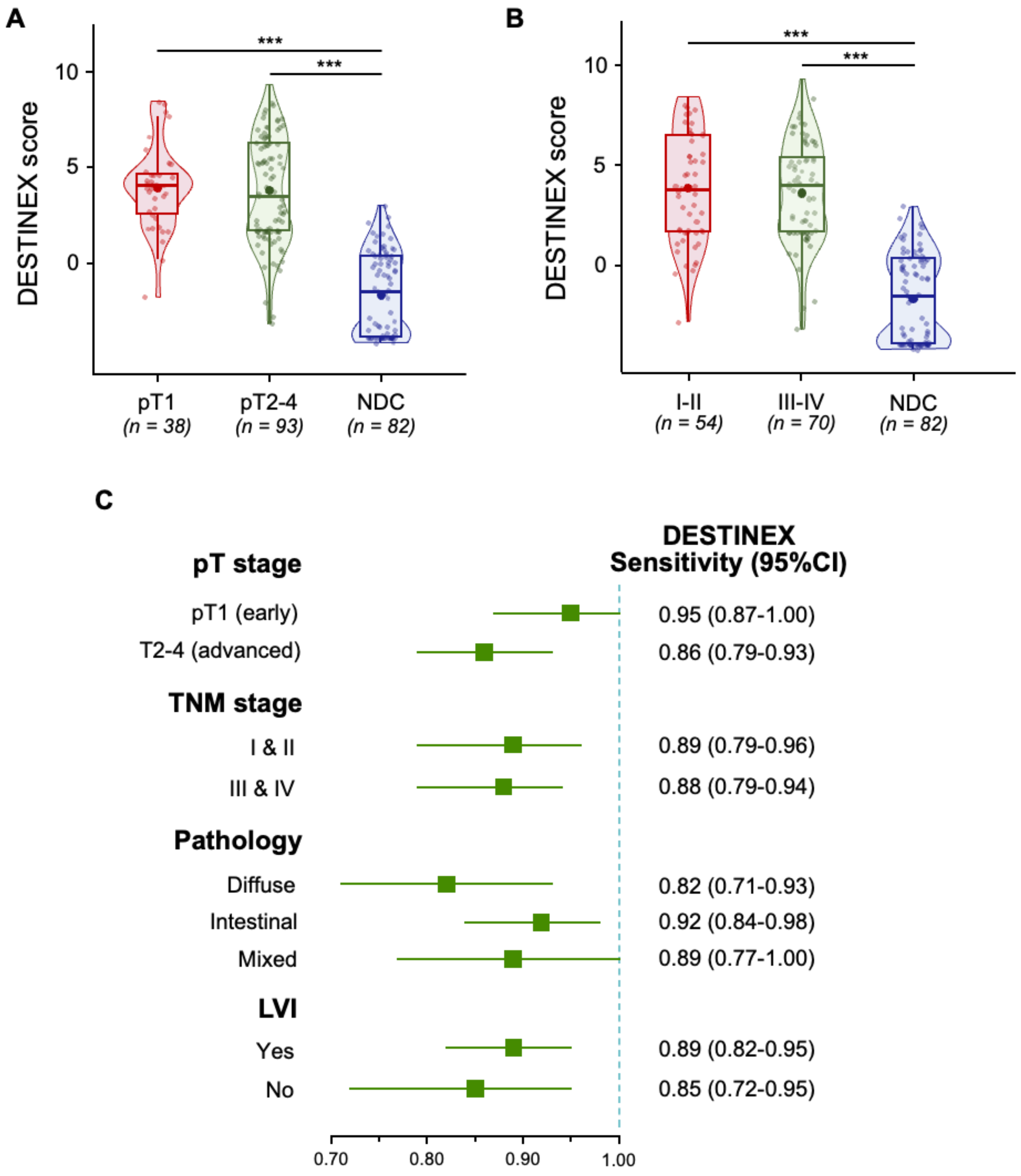

eFigure 5. The non-invasive DESTINEX efficiently identifies patients with early GC.

A. Risk scores calculated from DESTINEX are analyzed in NDCs, early-stage, and advanced-stage GC patients from the validation cohort. **B.** Risk score levels of DESTINEX in NDCs, I-II stage, and III-IV stage GC patients from the validation cohort. **C.** The green-filled squares represent point estimates of sensitivity, and horizontal lines indicate the 95% CIs. *** $p < 0.001$

© 2025 Sui S et al. *JAMA Surgery*.

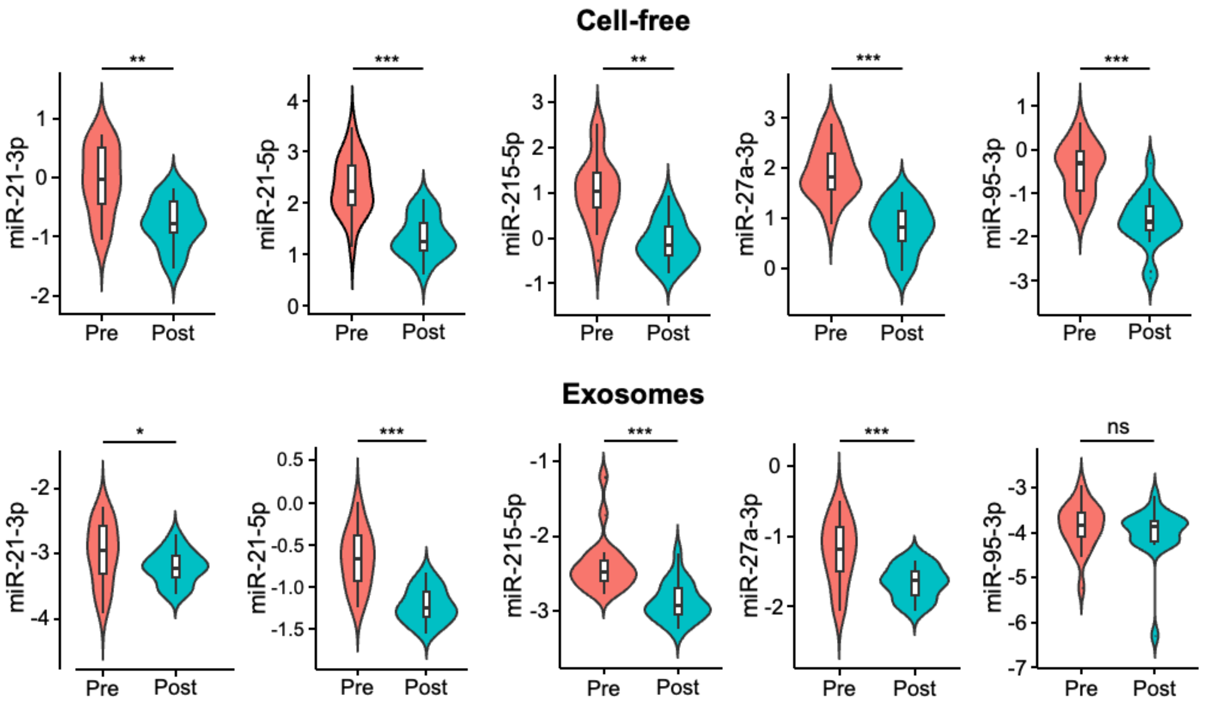

eFigure 6. The 10 individual miRNAs exhibited robust specificity for detecting GC. Expression levels of miRNA candidates in pre- and post-surgery serum samples from an independent cohort. * $p < 0.05$ ** $p < 0.01$ *** $p < 0.001$ ns: not significant

© 2025 Sui S et al. *JAMA Surgery.*

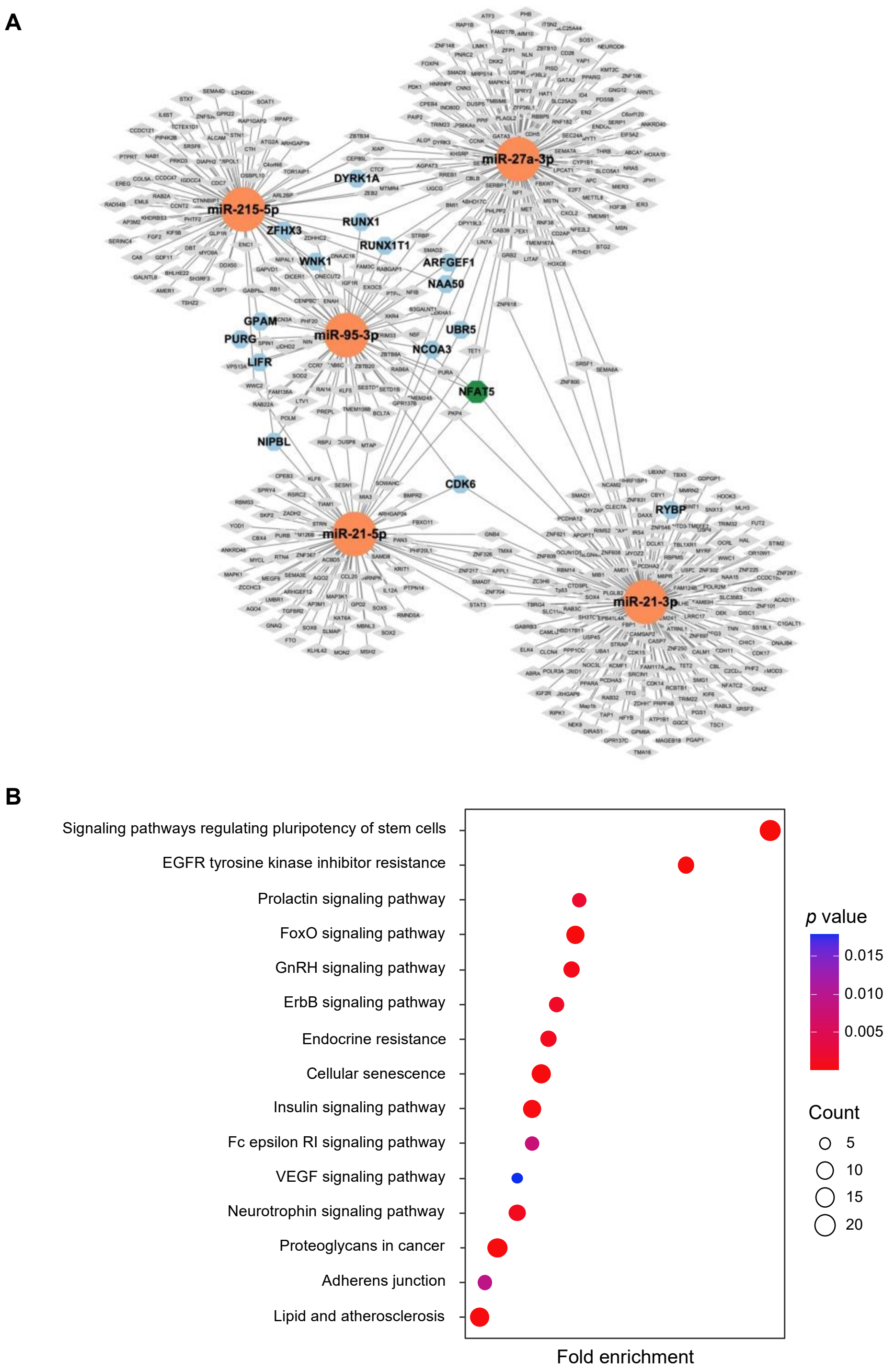

eFigure 7. Functional analysis of miRNA-target interactions and enriched pathways. **A.** Regulatory network of 5 miRNAs (miR-21-3p, miR-21-5p, miR-27a-3p, miR-95-3p, and miR-215-5p) and their predicted target genes. Nodes in orange represent hub miRNAs, gray nodes indicate predicted target genes, and green nodes denote key targets involved in cancer-related pathways. **B.** KEGG pathway enrichment analysis of predicted target genes.

© 2025 Sui S et al. *JAMA Surgery*.

eTable 1. Clinicopathological characteristics of enrolled patients in the training and validation cohorts.

	Training cohort (n = 263)		Validation cohort (n = 217)	
	**Patients with GC**	**Non-disease controls**	**Patients with GC**	**Non-disease controls**
Case number, n (%)	161 (61.2%)	102 (38.8%)	131 (60.4%)	86 (39.6%)
Age, median ± SD	68.4 ± 10.6	59.4 ± 10.2	61.5 ± 10.8	53.2 ± 4.8
Gender, n (%)				
Male	120 (74.5%)	69 (67.6%)	93 (80.0%)	54 (62.8%)
Female	41 (25.5%)	33 (32.4%)	31 (23.7%)	32 (37.2%)
NA	0 (0%)	0 (0%)	7 (5.3%)	0 (0%)
Tumor stage, n (%)				
I	76 (47.2%)	-	18 (13.7%)	-
II	29 (18.0%)	-	36 (27.5%)	-
III	47 (29.2%)	-	52 (39.7%)	-
IV	9 (5.6%)	-	18 (13.8%)	-
NA	0 (0%)	-	7 (5.3%)	-
CEA, median ± SD (ng/mL)	3.3 ± 3.4	-	4.2 ± 4.8	-

GC: Gastric cancer

CEA: Carcinoembryonic antigen

NA: Not available

SD: Standard deviation

© 2025 Sui S et al. *JAMA Surgery*.

eTable 2. Summary of the diagnostic performance of individual cell-free miRNAs in the training cohort.

miRNA, %	AUC (95%CI)	Sensitivity (95%CI)	Specificity (95%CI)	Accuracy (95%CI)	PPV (95%CI)	NPV (95%CI)
miR-21-3p	81.0 (75.9-86.1)	59.4 (51.8-67.0)	91.2 (85.7-96.7)	71.8 (71.6-71.9)	91.3 (85.9-96.7)	58.9 (51.2-66.5)
miR-21-5p	75.3 (69.5-81.2)	63.7 (56.3-71.2)	76.5 (68.2-84.7)	68.7 (68.5-68.9)	81.0 (74.1-87.8)	57.4 (49.0-65.7)
miR-215-5p	67.4 (60.8-74.0)	84.4 (78.7-90.0)	42.2 (32.6-51.7)	67.9 (67.8-68.1)	69.6 (63.1-76.1)	63.2 (51.8-74.7)
miR-27a-3p	74.8 (69.0-80.7)	54.4 (46.7-62.1)	84.3 (77.3-91.4)	66.0 (65.9-66.2)	84.5 (77.5-91.5)	54.1 (46.3-61.8)
miR-95-3p	71.1 (64.7-77.4)	75.5 (68.7-82.3)	56.6 (46.8-66.3)	68.1 (67.9-68.3)	73.1 (66.3-80.0)	59.6 (49.7-69.5)
miR-335-3p	85.5 (81.1-89.8)	67.5 (60.2-74.8)	92.2 (86.9-97.4)	77.1 (77.0-77.2)	93.1 (88.5-97.7)	64.4 (56.6-72.2)
miR-181b-5p	88.0 (84.0-92.0)	73.8 (66.9-80.6)	87.1 (80.6-93.7)	78.9 (78.8-79.1)	90.1 (85.0-95.2)	67.7 (59.7-75.7)
miR-431-5p	78.7 (73.3-84.1)	47.5 (39.8-55.2)	95.8 (91.8-99.8)	65.6 (65.5-65.8)	95.0 (90.2-99.8)	52.3 (44.9-59.7)

AUC: Area under the curve

CI: Confidence interval

PPV: Positive predictive value

NPV: Negative predictive value

© 2025 Sui S et al. *JAMA Surgery*.

eTable 3. Summary of the diagnostic performance of individual exosomal miRNAs in the training cohort.

miRNA, %	AUC (95%CI)	Sensitivity (95%CI)	Specificity (95%CI)	Accuracy (95%CI)	PPV (95%CI)	NPV (95%CI)
miR-21-3p	76.6 (70.9-82.2)	70.1 (62.9-77.2)	71.6 (62.8-80.3)	70.7 (70.5-70.8)	79.1 (72.4-85.9)	60.8 (52.1-69.6)
miR-21-5p	68.2 (61.8-74.6)	46.5 (38.8-54.3)	98.0 (95.3-100.0)	66.7 (66.5-66.8)	97.4 (93.8-100.0)	54.1 (46.9-61.2)
miR-215-5p	79.9 (74.5-85.3)	65.8 (58.4-73.2)	81.4 (73.8-88.9)	71.9 (71.8-72.1)	84.6 (78.2-90.9)	60.6 (52.4-68.8)
miR-27a-3p	69.0 (62.7-75.3)	46.2 (38.4-54.0)	96.1 (92.3-99.8)	65.8 (65.6-65.9)	94.8 (89.8-99.8)	53.6 (46.3-60.8)
miR-95-3p	63.4 (56.6-70.1)	51.6 (43.8-59.4)	75.5 (67.1-83.8)	61.0 (60.8-61.2)	76.4 (68.3-84.5)	50.3 (42.4-58.2)
miR-1246	60.2 (53.2-67.2)	31.4 (24.2-38.7)	87.3 (80.8-93.7)	53.3 (53.1-53.4)	79.4 (69.4-89.4)	44.9 (38.0-51.9)
miR-135b-5p	75.0 (67.4-82.6)	69.4 (54.4-84.5)	73.0 (67.2-78.8)	72.5 (72.4-72.7)	70.9 (80.5-61.3)	93.8 (90.2-97.3)
miR-183-5p	69.2 (62.8-75.7)	47.4 (39.4-55.3)	91.1 (85.5-96.6)	64.8 (64.6-65.0)	88.9 (82.0-95.7)	53.5 (46.0-60.9)
miR-196a-5p	70.0 (63.4-76.6)	85.4 (79.7-91.2)	47.5 (37.8-57.3)	69.8 (69.6-70.0)	69.9 (63.1-76.7)	69.6 (58.7-80.4)

AUC: Area under the curve

CI: Confidence interval

PPV: Positive predictive value

NPV: Negative predictive value

© 2025 Sui S et al. *JAMA Surgery*.

eTable 4. The diagnostic performance of *DESTINEX* at varying sensitivity thresholds for detection of pT1 GC.

	**AUC (95%CI)**	**Sensitivity (95%CI) @cutoff (1.065)[a]**	**Spec @90%Sens (95%CI)**	**Spec @92.5%Sens (95%CI)**	**Spec @95%Sens (95%CI)**
**Early stages (pT1), %**	96.8 (93.5-100.0)	95.0 (87.0-100.0)	93.9 (64.6-98.8)	90.2 (47.6-98.8)	73.2 (41.5-97.6)
**Late stages (pT2-4), %**	93.9 (90.7-97.2)	86.0 (79.0-93.0)	82.9 (59.8-93.9)	69.5 (50.0-91.5)	63.4 (41.5-85.4)

Sens: Sensitivity; **Spec**: Specificity; **a:** The cutoff value was determined from the training cohort.

© 2025 Sui S et al. *JAMA Surgery*.

eTable 5. Clinicopathological characteristics of the 20 patients with paired before and after surgery blood samples.

	**20 GC patients with paired pre- and post-surgery blood samples**
Age, median ± SD	58.5 ± 11.7
Gender, n (%)	
Male	14 (70.0%)
Female	6 (30.0%)
Tumor stage, n (%)	
I	8 (40.0%)
II	8 (40.0%)
III	4 (20.0%)

GC: Gastric cancer

SD: Standard deviation

© 2025 Sui S et al. *JAMA Surgery*.
