# Supplementary material for: Exosomal Liquid Biopsy for the Early Detection of Gastric Cancer: The DESTINEX Multicenter Study
Source: JAMA Surg. 2025 Jul 30;160(9):973–82. doi: 10.1001/jamasurg.2025.2493 (PMC12311825; doi:10.1001/jamasurg.2025.2493)
Supplement: Supplement 2. — Data Sharing Statement. [file jamasurg-e252493-s002.pdf]

Data Sharing Statement

Sui. Exosomal Liquid Biopsy for the Early Detection of Gastric Cancer. *JAMA Surg*. Published July 30, 2025. doi:10.1001/jamasurg.2025.2493

Data
Data available: Yes
Data types: Deidentified participant data
How to access data: Will be provided by email on request
When available: With publication

Supporting Documents
Document types: None

Additional Information
Who can access the data: None
Types of analyses: All data
Mechanisms of data availability: N/A
Any additional restrictions: N/A
